# U-Shaped Relationship of Low-Density Lipoprotein Cholesterol With Risk of Severe COVID-19 From a Multicenter Pooled Analysis

**DOI:** 10.3389/fcvm.2021.604736

**Published:** 2021-08-24

**Authors:** Jiao Gong, Yaqiong Chen, Yusheng Jie, Mingkai Tan, Zhaofang Jiang, Lianxiong Yuan, Jing Cao, Ganwen Li, Yutian Chong, Jiuxin Qu, Yaling Shi, Bo Hu

**Affiliations:** ^1^Department of Laboratory Medicine, Third Affiliated Hospital of Sun Yat-Sen University, Guangzhou, China; ^2^Department of Infectious Diseases, Key Laboratory of Liver Disease of Guangdong Province, Third Affiliated Hospital of Sun Yat-Sen University, Guangzhou, China; ^3^Department of Infectious Diseases, The Third Affiliated Hospital of Sun Yat-Sen University Yuedong Hospital, Meizhou, China; ^4^Department of Laboratory Medicine, Guangzhou Eighth People's Hospital, Guangzhou, China; ^5^Department of Clinical Laboratory, The Third People's Hospital of Shenzhen, Shenzhen, China; ^6^Department of Science and Research, Third Affiliated Hospital of Sun Yat-Sen University, Guangzhou, China

**Keywords:** lipid-lowering therapy, SARS-CoV-2, prognosis, severe COVID-19, LDL-C

## Abstract

Low-density lipoprotein cholesterol (LDL-C) is a well-known risk factor for coronary heart disease but protects against infection and sepsis. We aimed to disclose the exact association between LDL-C and severe 2019 novel coronavirus disease (COVID-19). Baseline data were retrospectively collected for 601 non-severe COVID-19 patients from two centers in Guangzhou and one center in Shenzhen, and patients on admission were medically observed for at least 15 days to determine the final outcome, including the non-severe group (*n* = 460) and the severe group (severe and critical cases) (*n* = 141). Among 601 cases, 76 (12.65%) received lipid-lowering therapy; the proportion of patients taking lipid-lowering drugs in the severe group was higher than that in the non-severe group (22.7 vs. 9.6%). We found a *U*-shaped association between LDL-C level and risk of severe COVID-19 using restricted cubic splines. Using univariate logistic regression analysis, odds ratios for severe COVID-19 for patients with LDL-C ≤1.6 mmol/L (61.9 mg/dL) and above 3.4 mmol/L (131.4 mg/dL) were 2.29 (95% confidence interval 1.12–4.68; *p* = 0.023) and 2.02 (1.04–3.94; *p* = 0.039), respectively, compared to those with LDL-C of 2.81–3.40 mmol/L (108.6–131.4 mg/dL); following multifactorial adjustment, odds ratios were 2.61 (1.07–6.37; *p* = 0.035) and 2.36 (1.09–5.14; *p* = 0.030). Similar results were yielded using 0.3 and 0.5 mmol/L categories of LDL-C and sensitivity analyses. Both low and high LDL-C levels were significantly associated with higher risk of severe COVID-19. Although our findings do not necessarily imply causality, they suggest that clinicians should pay more attention to lipid-lowering therapy in COVID-19 patients to improve clinical prognosis.

## Introduction

At present, the severe acute respiratory syndrome coronavirus 2 (SARS-CoV-2) has infected more than 21 million people worldwide. The COVID-19 pandemic has caused a global public health emergency. So far, no specific and effective therapeutic drugs are available for clinical management of COVID-19 patients. Thus, great interest is placed on identifying risk factors for preventing progression to severe COVID-19.

In patients with COVID-19, sepsis has been reported as the most frequently observed complication (1–3), and nearly 20% of the dead experienced secondary infections (4). Although low-density lipoprotein cholesterol (LDL-C) is a well-known risk factor for coronary heart disease, until recently, the association between low LDL-C and high risk of infection or sepsis was gradually uncovered. However, the exact association between LDL-C and severity of disease in COVID-19 is still unclear. Uncovering the association between LDL-C and severe COVID-19 is important—proprotein convertase subtilisin/kexin type 9 [PCSK9] inhibitors, a new medication, can significantly decrease LDL-C levels, which might greatly affect prognosis of COVID-19 patients.

Low LDL-C is a risk factor of infection, sepsis, and poor clinical outcome by epidemiological studies (5, 6). Low-density lipoproteins (LDLs) can attenuate sepsis-related lipopolysaccharide (LPS) damage (7) by binding to LPS (6) and thus lowering LPS-induced mortality *in vivo* studies (8, 9) and sepsis in human studies (6); LDL is a carrier for exogenous coenzyme Q10 (CoQ), which is a free radical scavenger with inhibiting arachidonic acid metabolic pathways, including the formation of various prostaglandins. LDL-C was decreased in COVID-19 patients (10, 11), implying its protective impact on COVID-19 progression and poor prognosis.

The study on association between LDL-C and severe COVID-19 is limited. In this paper, we analyzed the association between LDL-C and severe COVID-19 using restricted cubic splines, univariate and multivariate logistic regression analysis, and sensitivity analyses. We found that both low and high LDL-C levels were associated with severe COVID-19; 12.65% of COVID-19 patients received lipid-lowering therapy, and the proportion of patients taking lipid-lowering drugs in the severe group was higher than that in the non-severe group. Our findings suggests that clinicians should pay more attention to lipid-lowering therapy in COVID-19 patients to improve clinical prognosis, and a therapeutic strategy involving administering a certain concentration of lipoproteins to patients at greater risk for severe COVID-19 could potentially improve disease prognosis.

## Methods

### Data Collection

The data of 601 patients with laboratory-confirmed COVID-19, admitted to the Third Affiliated Hospital of Sun Yat-sen University, Guangzhou Eighth People's Hospital and The Third People's Hospital of Shenzhen from January 20, 2020, to April 25, 2020, were retrospectively analyzed. All included patients were medically observed at least 15 days after admission to determine whether they progressed to severe COVID-19 (severe group) or non-severe COVID-19 (non-severe group). Diagnostic criteria for SARS-CoV-2 infection have been described previously in a previous study (12). The diagnosis of severe COVID-19 was based on the Guidelines for Diagnosis and Treatment of Novel Coronavirus Pneumonia (6th version) released by the National Health Commission of China. Severe cases should meet at least one of the following criteria: (1) shortness of breath, respiratory rate (RR) ≥30 times/min; (2) arterial oxygen saturation (resting status) ≤ 93%; or (3) the ratio of partial pressure of oxygen to fraction of inspiration O_2_(PaO_2_/FiO_2_) ≤ 300 mmHg. Critical cases of COVID-19 should meet any of the following criteria: (1) respiratory failure requiring mechanical ventilation, (2) shock, and (3) with other organ failure that requires ICU care.

Clinical laboratory test results were collected from routine clinical practice. Written informed consent was waived by the Ethics Commission of each hospital for emerging infectious diseases. The study was approved by the Ethics Committee of the Eighth People's Hospital of Guangzhou, the Ethics Commission of the Third Affiliated Hospital of Sun Yat-sen University and the Ethics Commission of The Third People's Hospital of Shenzhen.

### Laboratory Methods

Clinical laboratory test results, including biochemical indexes and blood routine results, were obtained from routine clinical practice. The clinical laboratory examination results included the following: C-reactive protein (CRP, CRP-1003, Shenzhen Lifotronic Technology, Shenzhen, P.R. China), lactate dehydrogenase (LDH, 05169330190, Roche, Switzerland), blood urea nitrogen (BUN, 05171873190, Roche, Switzerland), cholesterol (CHOL, 05168538190, Roche, Switzerland), triglyceride (TG, 0517407190, Roche, Switzerland), high-density lipoprotein cholesterol (HDL-C, 07528582190, Roche, Switzerland), low-density lipoprotein cholesterol (LDL-C, 07005768190, Roche, Switzerland), albumin (ALB, 05166861190, Roche, Switzerland), white blood cell (WBC, SYSMEX, Japan), lymphocyte count (SYSMEX, Japan), neutrophil count (SYSMEX, Japan), and neutrophil-to-lymphocyte ratio (NLR). All parameters were obtained through standard automated laboratory methods with commercially available kits. The systems used in each laboratory were consistent.

### Statistical Analysis

Continuous variables and categorical variables were expressed and analyzed as previously described (12). Continuous variables were expressed as median (interquartile range [IQR]), or mean (standard deviation [SD]), as appropriate. Categorical variables were expressed as frequency and percentage. The difference between groups was analyzed using Fisher's exact test, parametric test (*t*-test), and non-parametric test (Mann–Whitney *U* test) for categorical variables and continuous variables with or without normal distribution, respectively. Except for filling in missing values, other statistical analyses were analyzed using R (version 3.6.2) with default parameters.

To minimize the bias of the regression coefficient, variable exclusion was limited to those with more than 1.5% missing rate. Little's MCAR test (R package BaylorEdPsych) was used to help assess the suitability of the remaining missing values for imputation as previously described (12). The missing values were imputed based on the expectation-maximization (EM) method using SPSS statistical software, version 25 (SPSS, Inc., Chicago, IL, USA).

The association between risk of severe COVID-19 and LDL-C and HDL-C on a continuous scale was examined using restricted cubic splines based on literature (13). These splines were used to help define reference groups using LDL-C categories, with the lowest risk category being the reference group. Patients with COVID-19 were also divided into five clinically relevant LDL-C groups with 0.5 mmol/L (19.3 mg/dL) and 0.6 mmol/L (23.2 mg/dL) intervals, and into eight groups with 0.3 mmol/L (11.6 mg/dL) intervals, respectively.

Restricted cubic splines and univariate and multivariate logistic regression analyses were used to identify risk factors that affect clinical outcomes by R software (R package: Hmisc, glmnet). The number of knots used in the restricted cubic spline was three. The knots were located at close to 1.6, 2.5, and 3.5, respectively. Sensitivity analyses in the cohort with LDL-C level were included by additionally adjusting for underlying disease, hypolipidemic drugs, and HDL-C and BMI levels. *p*-value < 0.05 was considered statistically significant.

## Results

### Clinicopathological Characteristics of Patients

**Figure 1:**
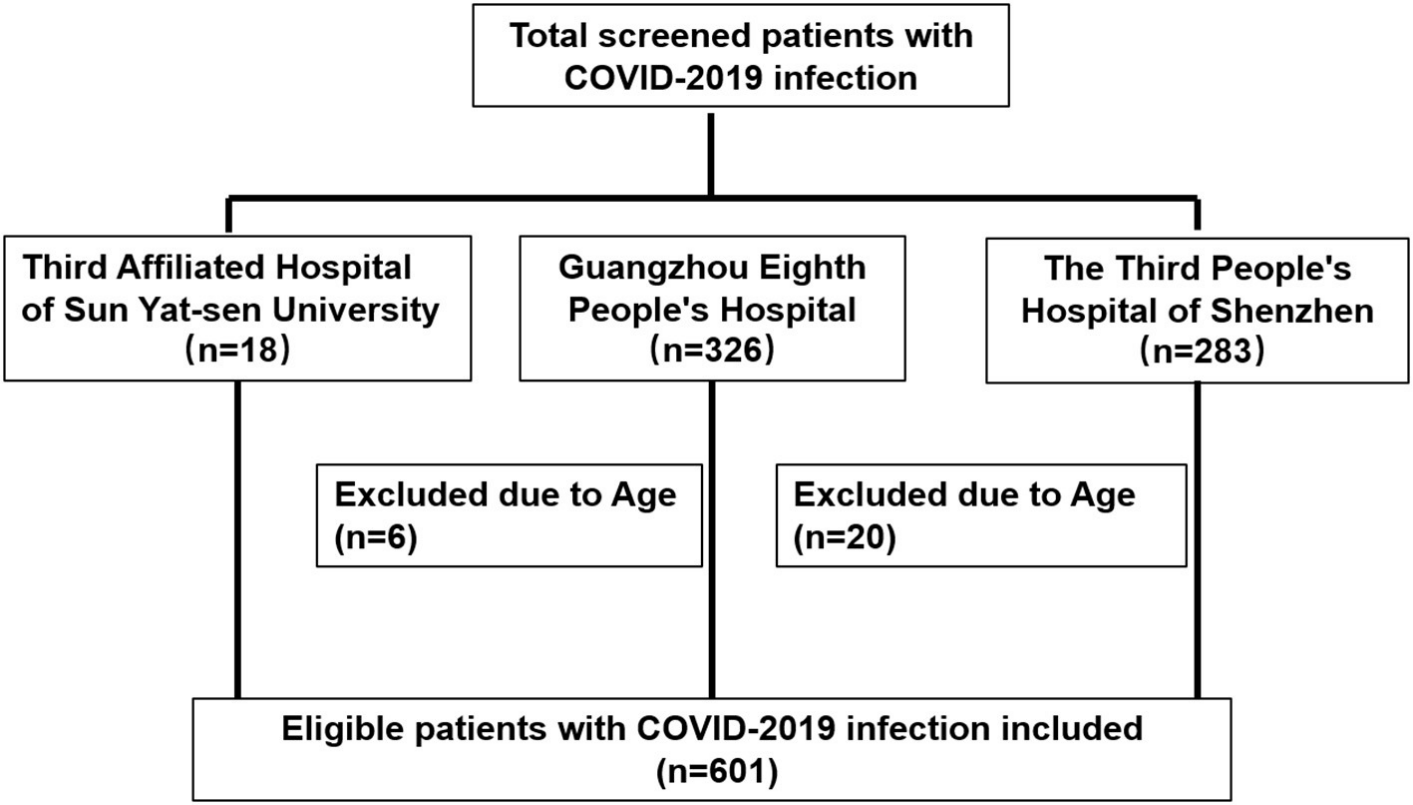
Flowchart of study participants in cohort.

The selection of the study population is shown in Figure 1. Of the 601 patients with non-severe COVID-19 included in this study on admission, 460 did not progress to severe COVID-19 (non-severe group) while 141 progressed to severe COVID-19 (severe group). The median age of the two groups was statistically different; the median age of the non-severe group was 41.5 years old, and that of the severe group was 60 years old. There was statistical difference in sex, fever, and underlying disease between the two groups at baseline, but the severe group consisted of more elderly patients than the non-severe group (*p* < 0.01) (Table 1). Among 601 cases, 287 (47.75%) cases presented with fever, 28 (4.66%) cases presented with diarrhea, 158 (26.29%) cases had underlying diseases, and 76 (12.65%) received lipid-lowering therapy. There was no significant difference in WBC, serum CHOL, and LDL-C level but HDL-C at baseline between severe and non-severe groups. However, when the severe group was further stratified into severe and critical group, the LDL-C level was significantly lower in critical cases (Supplementary Table 1).

**Table 1:** Baseline characteristics of COVID-19 patients.

	**Non-severe** **(*n* = 460)**	**Severe** **(*n* = 141)**	***p-*value**
Age (year)	41.5 (31.0, 55.0)	60.0 (50.0, 65.0) (*n* = 141)	<0.01
Sex			<0.01
Female	244 (53.0%)	51 (36.2%)	
Male	216 (47.0%)	90 (63.8%)	
Basic disease			<0.01
No	362 (78.7%)	81 (57.4%)	
Yes	98 (21.3%)	60 (42.6%)	
BMI, median	23.2 (21.0, 25.5) (*n* = 459)	24.2 (22.8, 26.6) (*n* = 138)	<0.01
Hypolipidemic drugs			<0.01
No	416 (90.4%)	109 (77.3%)	
Yes	44 (9.6%)	32 (22.7%)	
Fever			<0.01
No	270 (58.7%)	44 (31.2%)	
Yes	190 (41.3%)	97 (68.8%)	
Diarrhea			0.06
No	443 (96.3%)	130 (92.2%)	
Yes	17 (3.7%)	11 (7.8%)	
Coronary artery disease			0.08
No	446 (97.0%)	132 (93.6%)	
Yes	14 (3.0%)	9 (6.4%)	
Previous stroke			0.41
No	459 (99.8%)	140 (99.3%)	
Yes	1 (0.2%)	1 (0.7%)	
PAD			0.23
No	460 (100.0%)	140 (99.3%)	
Yes	0 (0.0%)	1 (0.7%)	
Atrial fibrillation			0.14
No	459 (99.8%)	139 (98.6%)	
Yes	1 (0.2%)	2 (1.4%)	
COPD			0.05
No	460 (100.0%)	139 (98.6%)	
Yes	0 (0.0%)	2 (1.4%)	
Smoke			0.29
No	235 (51.1%)	64 (45.4%)	
Yes	34 (7.4%)	5 (3.5%)	
Missing	191 (41.5%)	72 (51.1%)	
WBC (×10^9^/L)	4.83 (3.96, 5.96)	4.79 (4.02, 6.28)	0.7
Neutrophils (×10^9^/L)	2.81 (2.14, 3.72) (*n* = 459)	3.15 (2.34, 4.40) (*n* = 141)	<0.01
Lymphocyte (×10^9^/L)	1.41 (1.07, 1.88)	1.02 (0.83, 1.40)	<0.01
NLR	1.92 (1.45, 2.90) (*n* = 459)	2.92 (1.89, 4.78) (*n* = 141)	<0.01
INR	1.02 (0.98, 1.07) (*n* = 266)	1.04 (0.99, 1.10) (*n* = 67)	0.09
ALB (g/L)	42.85 (40.20, 45.50) (*n* = 458)	38.95 (35.45, 42.95) (*n* = 140)	<0.01
CRP (mg/L)	5.00 (5.00, 14.84) (*n* = 456)	28.10 (9.10, 52.10) (*n* = 141)	<0.01
BUN (mmol/L)	3.69 (3.05, 4.50) (*n* = 323)	4.80 (3.42, 6.36) (*n* = 115)	<0.01
LDH (U/L)	179.00 (153.00, 222.50) (*n* = 452)	268.50 (198.00, 440.00) (*n* = 140)	<0.01
TG (mmol/L)	1.09 (0.76, 1.62) (*n* = 458)	1.19 (0.92, 1.64) (*n* = 141)	0.04
LDL-C (mmol/L)	2.54 (2.01, 3.08) (*n* = 458)	2.41 (1.91, 3.01) (*n* = 141)	0.23
CHOL (mmol/L)	4.17 (0.95) (*n* = 458)	4.12 (1.06) (*n* = 141)	0.63
HDL-C (mmol/L)	1.12 (0.91, 1.35) (*n* = 458)	1.02 (0.83, 1.25) (*n* = 141)	<0.01

*Features with missing values are labeled with a specific number of samples*.

*BMI, body mass index; WBC, white blood cell; NLR, neutrophil-to-lymphocyte ratio; INR, international normalized ratio; ALB, albumin; BUN, blood urea nitrogen; LDH, lactate dehydrogenase; CRP, C-reactive protein; TG, triglycerides; LDL-C, low-density lipoprotein cholesterol; CHOL, cholesterol; HDL-C, high-density lipoprotein cholesterol; PAD, peripheral arterial disorder; Basic disease, patients with one of the following: hypertension, diabetes, cardiovascular disease, chronic respiratory disease, or tuberculosis disease*.

### Relationship Between Low and High Serum LDL-C Levels (*U*-Shaped) and Risk of Severe COVID-19 Disease

**Figure 2:**
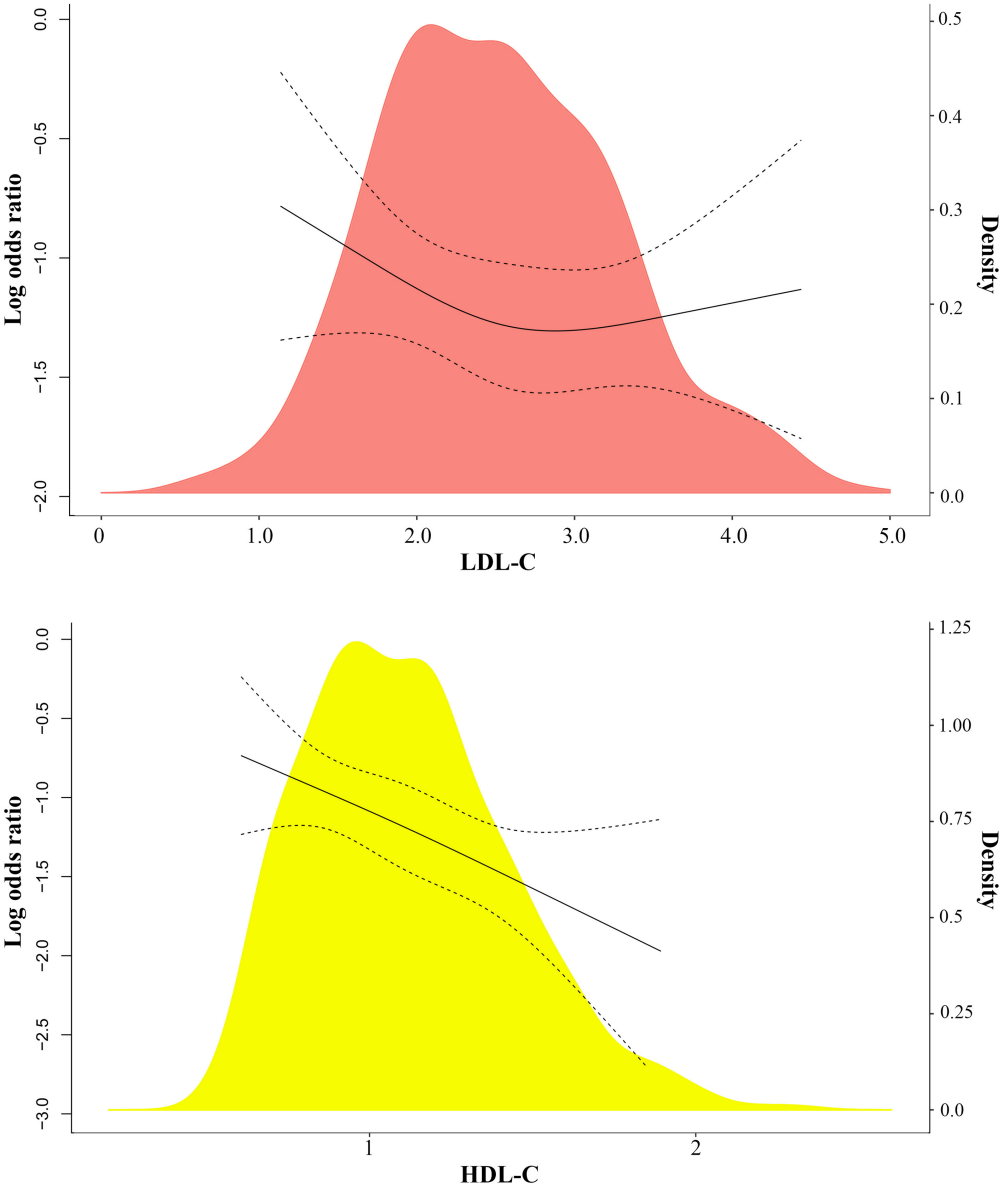
Association between LDL-C, HDL-C, and severe COVID-19 using restricted cubic splines. (A) LDL-C and HDL-C on a continuous scale and risk of severe COVID-19 in 601 patients with SARS-CoV-2. Analyses were conducted using restricted cubic splines. The red and yellow areas indicate the distribution of LDL-C and HDL-C, respectively.

**Figure 3:**
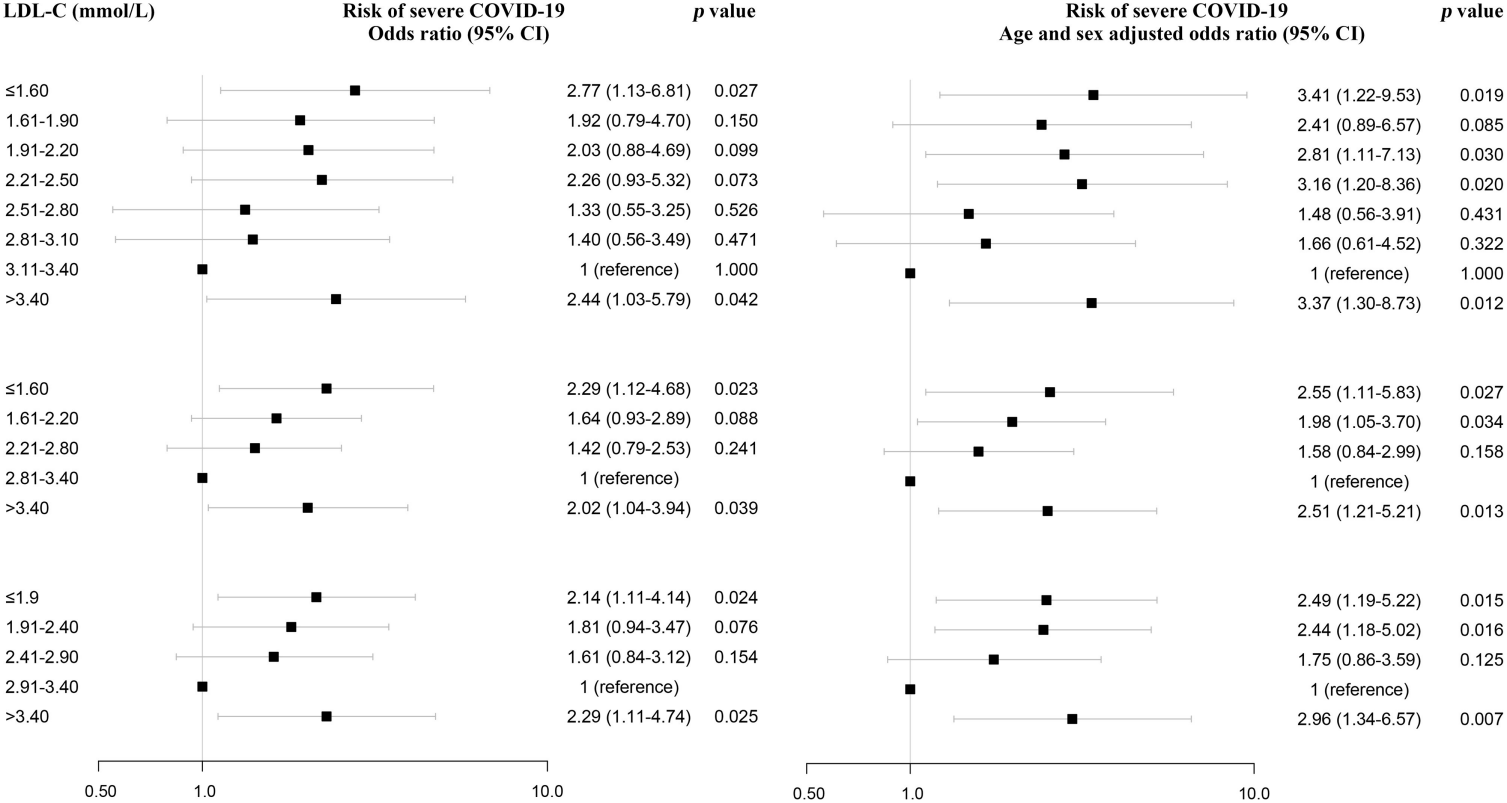
Association between low and high serum LDL-C levels and severe COVID-19 from univariate logistic regression analysis. LDL-C and risk of severe COVID-19. Individuals are in the top part divided into 0.3 mmol/L (11.6 mg/dL) intervals, and in the bottom part into 0.6 mmol/L (23.2 mg/dL) and 0.5 mmol/L (19.3 mg/dL) intervals of LDL-C. The values of LDL-C with the lowest odds ratio were chosen as reference. Odds ratios were from univariate or multivariate logistic regression analysis.

Using restricted cubic splines, both low and high serum LDL-C level (*U*-shaped) were associated with a high risk of severe COVID-19 (Figure 2; Supplementary Figure 1A). High HDL-C levels were associated with low risk of severe COVID-19 (Figure 2). When LDL-C levels were divided into 0.3 mmol/L (11.6 mg/dL) intervals, the risk of severe COVID-19 showed a *U*-shaped pattern with high risk associated with both low and very high levels of LDL-C, compared with the reference group with LDL-C levels (3.11–3.40 mmol/L) (120.2–131.4 mg/dL) (Figure 3, upper left part). Non-severe patients with LDL-C ≤1.6 mmol/L (61.9 mg/dL) had an age- and gender-adjusted odds ratio for severe disease of 3.41 (95% confidence interval 1.22–9.53) as compared to those with LDL-C level (3.11–3.40 mmol/L; 120.2–131.4 mg/dL) (Figure 3, upper right part). Non-severe patients with high LDL-C levels (>3.4 mmol/L; 131.4 mg/dL) also showed high risk of severe COVID-19 with a corresponding odds ratio of 3.37 (1.30–8.73).

Consistent with the above results, there was an U-shaped association between LDL-C and risk of severe COVID-19 when serum LDL-C levels were divided into 0.6 mmol/L (23.2 mg/dL) intervals and 0.5 mmol/L (19.3 mg/dL) intervals (Figure 3, lower left part). Even after adjusting for age and gender, this association persisted, with an odds ratio for severe COVID-19 of 2.55 (1.11–5.83) for cases with LDL-C ≤1.6 mmol/L (61.9 mg/dL) compared to cases with LDL-C of 2.81–3.40 mmol/L; 108.6–131.4 mg/dL) using 0.6 intervals and 2.49 (1.19–5.22) for cases with LDL-C ≤1.9 mmol/L (73.5 mg/dL) compared to cases with LDL-C of 2.91–3.40 mmol/L (Figure 3, lower right part).

### Univariate and Multivariate Logistic Regression Analysis

**Figure 4:**
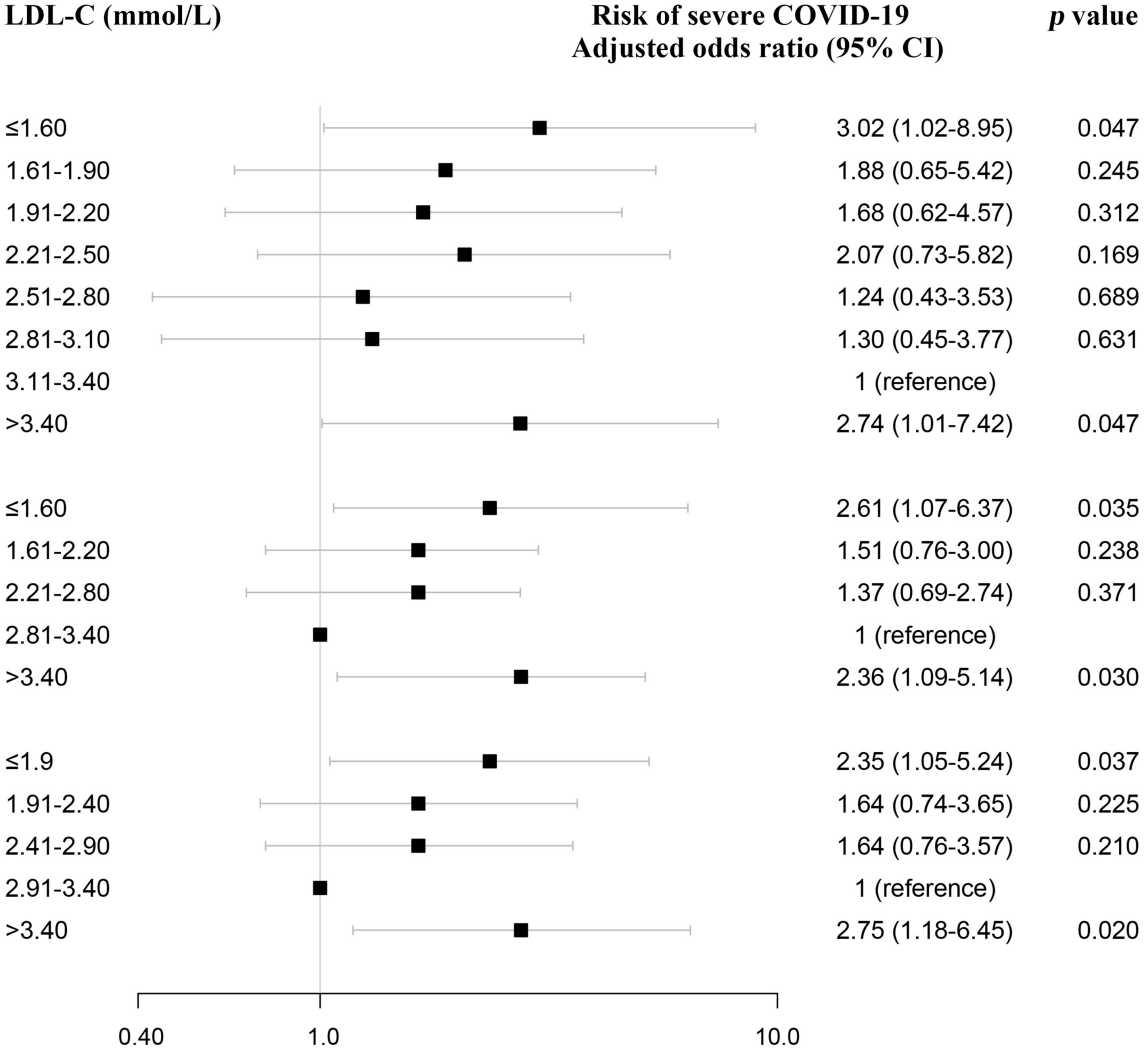
Association between LDL-C and severe COVID-19 using multivariate logistic regression analysis and sensitivity analysis. LDL-C and risk of severe COVID-19 using multivariate logistic regression analysis. After adjusting for age, gender, CRP, LDH, and NLR, odds ratios were from multivariate logistic regression analysis. CRP, C-reactive protein; LDH, lactate dehydrogenase; NLR, neutrophil-to-lymphocyte ratio.

Furthermore, variables associated with severe COVID-19 were analyzed by the univariate logistic regression analysis, and among them, old age, gender, high CRP, LDH, and NLR were significantly associated with severe COVID-19 (Supplementary Table 2). Then, these variables and LDL-C were included in a multivariate logistic regression analysis. The multivariate logistic regression analysis also demonstrated that LDL-C ≤1.6 mmol/L was a risk factor, with odds ratio of 3.02 (1.02–8.95) using 0.3 mmol/L categories of LDL-C (Figure 4; Supplementary Table 2). Similar results were obtained using 0.6 mmol/L and 0.5 mmol/L categories of LDL-C (Figure 4; Supplementary Table 3). Moreover, we analyzed the association between lipid-lowering therapy and severe COVID-19 after stratifying the analyses based on LDL-C. The high percentage of severe COVID-19 was observed in patients with lipid-lowering therapy for LDL-C below 3.4 (Supplementary Table 4). In addition, using restricted cubic splines, results were similar when adjusting for CRP, LDH, and NLR (Supplementary Figure 1B).

### Sensitivity Analyses

**Figure 5:**
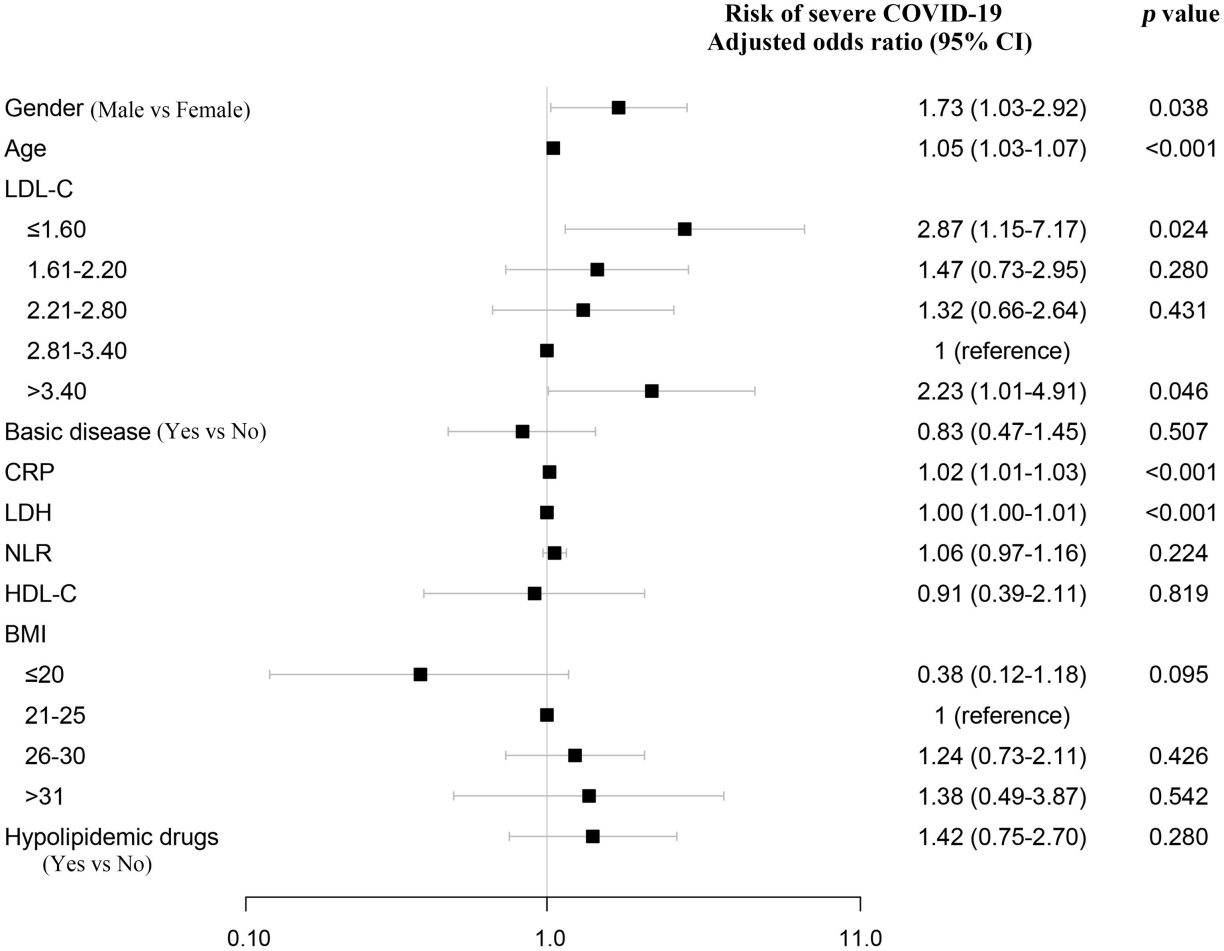
Sensitivity analysis. Sensitivity analysis that additionally adjusted for age, gender, basic disease, CRP, LDH, NLR, HDL-C, BMI, and hypolipidemic drugs. Odds ratios were from multivariate logistic regression analysis. NLR, neutrophil-to-lymphocyte ratio.

The association between both low and high serum LDL-C level (*U*-shaped) and high risk of severe COVID-19 was present after adjusting for age, gender, underlying disease, CRP, LDH, NLR, HDL-C, BMI, and hypolipidemic drugs (Figure 5; Supplementary Figures 2, 3). As women on average have higher LDL-C than men, we also performed restricted cubic spline models in male and female separately, and observed a similar association between low LDL-C and high risk of severe COVID-19 (Supplementary Figure 4).

## Discussion

In this research, we observed that LDL-C levels were lower in critical COVID-19 patients than in non-severe and severe patients. More than 10% of COVID-19 patients received lipid-lowering therapy; the proportion of patients taking lipid-lowering drugs in the severe group was higher than that in the non-severe group (22.7 vs. 9.6%). Both low and high LDL-C levels on admission were associated with high risk of severe COVID-19. After adjusting for other variables, LDL-C was also an independent risk factor.

It is likely that the explanation for risk for severe COVID-19 disease differs in individuals with low and high LDL-C levels. Relevant for high LDL-C, hyperlipidemia can significantly increase a person's risk of coronary heart disease (CHD), stroke, and other serious problems, and lipid-lowering therapy can reduce the risk of cardiovascular events. Relevant for low LDL-C, it might contribute to the function of immune system by several possible mechanisms: (1) LDL-C levels are associated with hematopoietic stem cell (HSC) frequency in circulation (14). LDL-C upregulates the number of CD34^+^ hematopoietic stem and progenitor cells (HSPCs) through increasing proliferation as well as stimulating the level of mobilizing cytokines such as granulocyte colony-stimulating factor (G-CSF) and interleukin 17 (IL-17), thereby controlling the number of immune cells and HSPCs in the circulation (14). (2) LDL can bind toxic bacterial products such as LPS and prevent or improve many of the manifestations of sepsis *via* immunological effects (6). Increasing LDL-C levels protected LDL receptor-deficient mice from lethal endotoxemia or severe infections with Gram-negative microorganisms, and hypolipidemic mice could be rescued by administering exogenous lipoprotein to raise serum lipid levels to within the normal physiological range (9). (3) LDL is a carrier of exogenous coenzyme Q10 (CoQ), which is an effective agent for attenuating the harmful effects of septic shock. CoQ, a free radical scavenger, inhibits arachidonic acid metabolic pathways, including the formation of various prostaglandins (15). However, statins have been reported to decrease CoQ concentrations in blood (16) and might cause poor prognosis of COVID-19. These findings may explain our findings that low LDL levels were associated with the severity of COVID-19.

Although bacterial infections are generally considered to be the main cause of sepsis, viral infections can also cause sepsis. In patients with COVID-19, sepsis was the most frequently observed complication (2, 3), followed by respiratory failure, ARDS, heart failure, and septic shock (17). Low LDL-C levels were associated with increased risk of fever and sepsis in hospitalized patients, or death in community-acquired patients pneumonia (7, 18) and poor clinical outcomes of sepsis (19, 20). Healthy individuals with low LDL-C have a significantly increased risk of infectious diseases (21), not to mention individuals already with SARS-CoV-2 infection. Consistent with the above findings, we found that LDL-C was also an independent risk factor for severity of COVID-19 even after adjusting for other factors (age, gender, CRP, LDH, and NLR).

Impairment of lipid metabolism including low LDL-C was found as a major change in septic patients with hospital-acquired pneumonia by proteomic analysis (22). We similarly observed that LDL-C level was lower in critical COVID-19 patients than in non-severe and severe COVID-19 patients. The decreased LDL-C in COVID-19 patients may be due to the following reasons: (1) decreased plasma LDL levels were caused partially by tumor necrosis factor (TNFα) (7); (2) upregulation of interleukin 6 (IL-6) decreased LDL-C levels by stimulating the uptake of LDL particles (23). IL-6 and TNFα were significantly elevated in COVID-19 patients, which might result in low LDL-C level (1). The lower baseline LDL-C levels observed in critical COVID-19 patients support the finding that low LDL-C levels were associated with severe COVID-19.

Increasing age is a predisposing factor for dyslipidemia. Consistent with other studies, our research showed that old age was a risk factor for severity of COVID-19. In our research, 76 (12.65%) cases used hypolipidemic drugs, and the proportion of taking antihyperlipidemic drugs in the severe group was also higher than that in the non-severe group (22.7 vs. 9.6%). Our findings suggest that LDL-C ≤1.6 mmol/L is a risk factor for severe COVID-19. Hence, lipid-lowering therapy should be done cautiously in elderly patients with COVID-19, not only when they are in critical condition, but also when they are already at increased risk for severe COVID-19 disease. In future studies, it would be important to determine the association between lipid-lowering therapy and severe COVID-19 in elderly patients and how this affects prognosis.

The present study has several strengths. The data were obtained from three centers, and with a relatively large sample size. There were some limitations in the study. First, this is a retrospective study, including 601 patients with non-severe COVID-19 on admission. Second, we did not analyze the effect of LDL-C genetic risk score based on single-nucleotide polymorphism data. Third, LDL-C level has been reported to be differentially expressed in different populations (24). Hence, given the lipid-lowering therapy, the optimal LDL-C level in elderly COVID-19 patients may be different to decrease the higher odds of severe COVID-19. Fourth, the final survival outcome is lacking. Fifth, the study did not include the level of small dense LDL particles, which has been reported to be sensitive to oxidative damage, and increased in inflammation and infection (7). More investigations with large populations will need to be done to confirm the role of LDL-C in COVID-19.

## Conclusion

In summary, both low and high LDL-C levels are associated with risk of severe COVID-19 and lipid-lowering therapy should be done cautiously in elderly patients with COVID-19. Our findings highlighted that moderate LDL-C is better than high LDL-C, but extremely low LDL-C may be harmful. It must be emphasized that the association between LDL-C and severe COVID-19 does not necessarily indicate causality; however, our findings stress that more attention toward identifying the casual relationship between LDL-C and severe COVID-19 is much needed.

## Data Availability Statement

All data generated or analyzed during this study are included in this published article and its Supplementary Material.

## Ethics Statement

The studies involving human participants were reviewed and approved by the Ethics Committee of the Eighth People's Hospital of Guangzhou, the Ethics Commission of the Third Affiliated Hospital of Sun Yat-sen University and the Ethics Commission of The Third People's Hospital of Shenzhen. Written informed consent for participation was not required for this study in accordance with the national legislation and the institutional requirements.

## Author Contributions

BH, YS, and JQ designed the study and had full access to all data in the study and take responsibility for the integrity of the data and the accuracy of the data analysis. JG, YqC, YJ, MT, ZJ, and YtC contributed to collect data, analyze data, and write the paper. LY, JC, and GL contributed to the statistical analysis. All authors contributed to data interpretation and reviewed and approved the final version.

## Conflict of Interest

The authors declare that the research was conducted in the absence of any commercial or financial relationships that could be construed as a potential conflict of interest.

## Publisher's Note

All claims expressed in this article are solely those of the authors and do not necessarily represent those of their affiliated organizations, or those of the publisher, the editors and the reviewers. Any product that may be evaluated in this article, or claim that may be made by its manufacturer, is not guaranteed or endorsed by the publisher.
