# Supplementary material for: U-Shaped Relationship of Low-Density Lipoprotein Cholesterol With Risk of Severe COVID-19 From a Multicenter Pooled Analysis
Source: Front Cardiovasc Med. 2021 Aug 24;8:604736. doi: 10.3389/fcvm.2021.604736 (PMC8421675; doi:10.3389/fcvm.2021.604736)
Supplement: Supplementary file 1 [file Data_Sheet_1.docx]

Supplementary Table 1. Demographics and characteristics of COVID-19 patients

|  | Non-severe (n=460) | Severe (n=114) | Critical (n=27) | *p* value |
| --- | --- | --- | --- | --- |
| Age | 41.5 (31.0, 55.0) | 58.0 (46.0, 64.0) (n=114) | 65.0 (58.0, 69.0) (n=27) | <0.01 |
| Gender |  |  |  | <0.01 |
| Female | 244 (53.0%) | 44 (38.6%) | 7 (25.9%) |  |
| Male | 216 (47.0%) | 70 (61.4%) | 20 (74.1%) |  |
| Basic disease |  |  |  | <0.01 |
| No | 362 (78.7%) | 70 (61.4%) | 11 (40.7%) |  |
| Yes | 98 (21.3%) | 44 (38.6%) | 16 (59.3%) |  |
| BMI, median | 23.2 (21.0, 25.5) (n=459) | 24.3 (23.0, 26.7) (n=111) | 23.4 (21.5, 25.4) (n=27) | <0.01 |
| Hypolipidemic drugs |  |  |  | <0.01 |
| No | 416 (90.4%) | 87 (76.3%) | 22 (81.5%) |  |
| Yes | 44 (9.6%) | 27 (23.7%) | 5 (18.5%) |  |
| Exposure |  |  |  | <0.01 |
| No | 216 (47.0%) | 38 (33.3%) | 6 (22.2%) |  |
| Close contact with Wuhan | 244 (53.0%) | 76 (66.7%) | 21 (77.8%) |  |
| Fever |  |  |  | <0.01 |
| No | 270 (58.7%) | 37 (32.5%) | 7 (25.9%) |  |
| Yes | 190 (41.3%) | 77 (67.5%) | 20 (74.1%) |  |
| Diarrhea |  |  |  | 0.06 |
| No | 443 (96.3%) | 106 (93.0%) | 24 (88.9%) |  |
| Yes | 17 (3.7%) | 8 (7.0%) | 3 (11.1%) |  |
| WBC (x10^9^/L) | 4.83 (3.96, 5.96) | 4.62 (3.93, 5.85) | 5.41 (4.38, 7.00) | 0.05 |
| Neutrophils (x10^9^/L) | 2.81 (2.14, 3.72) (n=459) | 2.96 (2.25, 4.21) (n=114) | 4.04 (3.33, 5.35) (n=27) | <0.01 |
| Lymphocyte (x10^9^/L) | 1.41 (1.07, 1.88) | 1.06 (0.83, 1.40) | 0.92 (0.55, 1.35) | <0.01 |
| NLR | 1.92 (1.45, 2.90) (n=459) | 2.81 (1.83, 4.35) (n=114) | 3.86 (2.28, 5.90) (n=27) | <0.01 |
| INR | 1.02 (0.98, 1.07) (n=266) | 1.04 (0.99, 1.10) (n=57) | 1.06 (0.99, 1.16) (n=10) | 0.22 |
| ALB (g/L) | 42.85 (40.20, 45.50) (n=458) | 39.60 (37.00, 43.00) (n=113) | 35.10 (32.60, 39.60) (n=27) | <0.01 |
| CRP (mg/L) | 5.00 (5.00, 14.84) (n=456) | 26.64 (5.99, 51.70) (n=114) | 34.20 (20.44, 56.30) (n=27) | <0.01 |
| BUN (mmol/L) | 3.69 (3.05, 4.50) (n=323) | 4.52 (3.32, 5.99) (n=90) | 5.47 (4.48, 7.91) (n=25) | <0.01 |
| LDH (U/L) | 179.00 (153.00, 222.50) (n=452) | 268.00 (198.00, 412.00) (n=113) | 407.00 (205.00, 720.00) (n=27) | <0.01 |
| TG (mmol/L) | 1.09 (0.76, 1.62) (n=458) | 1.15 (0.92, 1.68) (n=114) | 1.25 (0.88, 1.59) (n=27) | 0.12 |
| LDL-C (mmol/L) | 2.54 (2.01, 3.08) (n=458) | 2.47 (1.99, 3.11) (n=114) | 2.09 (1.67, 2.54) (n=27) | 0.02 |
| CHOL (mmol/L) | 4.17 (0.95) (n=458) | 4.22 (1.06) (n=114) | 3.70 (0.97) (n=27) | 0.04 |
| HDL-C (mmol/L) | 1.12 (0.91, 1.35) (n=458) | 1.09 (0.85, 1.25) (n=114) | 0.88 (0.80, 1.06) (n=27) | <0.01 |

Features with missing values are labeled with a specific number of samples.

Abbreviations: BMI, body mass index; WBC, white blood cell; NLR, neutrophil-to-lymphocyte ratio; INR, international normalized ratio; ALB, albumin; BUN, blood urea nitrogen; LDH, lactate dehydrogenase; CRP, C-reactive protein; TG, triglycerides; LDL-C, low-density lipoprotein cholesterol; CHOL, cholesterol; HDL-C, high-density lipoprotein cholesterol.

Supplementary Table 2. Univariate and multivariate analysis of factors associated with severe COVID-19

| Variable | Odds ratio (95% CI) | *p* value |
| --- | --- | --- |
| **Univariate analysis** ^a^ | 1.07 (1.05-1.08) |  |
| Gender  (Male vs Female) |  | <0.001 |
| Age  (Per 1 year increase) | 1.99 (1.35-2.94) | 0.001 |
| LDL-C |  |  |
| ≤1.60 | 2.77 (1.13-6.81) | 0.027 |
| 1.61-1.90 | 1.92 (0.79-4.70) | 0.150 |
| 1.91-2.20 | 2.03 (0.88-4.69) | 0.099 |
| 2.21-2.50 | 2.26 (0.93-5.32) | 0.073 |
| 2.51-2.80 | 1.33 (0.55-3.25) | 0.526 |
| 2.81-3.10 | 1.40 (0.56-3.49) | 0.471 |
| 3.11-3.40 | 1 (reference) | 1.000 |
| >3.40 | 2.44 (1.03-5.79) | 0.042 |
| CRP | 1.04 (1.03-1.05) | <0.001 |
|  |  |  |
| LDH | 1.01 (1.00-1.01) | <0.001 |
| NLR | 1.27 (1.17-1.38) | <0.001 |
|  |  |  |
| **Multivariate analysis ^b^** |  |  |
| Gender | 2.02 (1.25-3.28) | 0.004 |
| Age | 1.05 (1.03-1.07) | <0.001 |
| LDL-C |  |  |
| ≤1.60 | 3.02 (1.02-8.95) | 0.047 |
| 1.61-1.90 | 1.88 (0.65-5.42) | 0.245 |
| 1.91-2.20 | 1.68 (0.62-4.57) | 0.312 |
| 2.21-2.50 | 2.07 (0.73-5.82) | 0.169 |
| 2.51-2.80 | 1.24 (0.43-3.53) | 0.689 |
| 2.81-3.10 | 1.30 (0.45-3.77) | 0.631 |
| 3.11-3.40 | 1 (reference) |  |
| >3.40 | 2.74 (1.01-7.42) | 0.047 |
| CRP | 1.02 (1.01-1.03) | <0.001 |
| LDH | 1.00 (1.00-1.01) | <0.001 |
| NLR | 1.05 (0.96-1.15) | 0.286 |

Abbreviations: LDH, lactate dehydrogenase; CRP, C-reactive protein; LDL-C, low-density lipoprotein cholesterol; 95% CI, 95% confidence interval.

a: Univariate analysis, univariate logistic regression analysis.

b: Multivariate analysis, multivariate logistic regression analysis

Supplementary Table 3. Multivariate analysis of factors associated with severe COVID-19

| Variable | Odds ratio (95% CI) | p value |
| --- | --- | --- |
| Gender (Male vs Female) | 2.03 (1.26-3.29) ^a^ | 0.004 ^a^ |
| Age (Per 1 year increase) | 1.05 (1.03-1.07) ^a^ | <0.001 ^a^ |
| LDL-C |  |  |
| ≤1.60 | 2.61 (1.07-6.37) ^a^ | 0.035 ^a^ |
| 1.61-2.20 | 1.51 (0.76-3.00) ^a^ | 0.238 ^a^ |
| 2.21-2.80 | 1.37 (0.69-2.74) ^a^ | 0.371 ^a^ |
| 2.81-3.40 | 1 (reference) |  |
| >3.40 | 2.36 (1.09-5.14) ^a^ | 0.030 ^a^ |
| CRP | 1.02 (1.01-1.03) ^a^ | <0.001 ^a^ |
| LDH | 1.00 (1.00-1.01) ^a^ | <0.001 ^a^ |
| NLR | 1.05 (0.96-1.15) ^a^ | 0.281 ^a^ |
|  |  |  |
|  |  |  |
| Gender (Male vs Female) | 2.01 (1.25-3.26) ^b^ | 0.004 ^b^ |
| Age (Per 1 year increase) | 1.05 (1.03-1.07) ^b^ | < 0.001 ^b^ |
| LDL-C |  |  |
| ≤1.9 | 2.35 (1.05-5.24) ^b^ | 0.037 ^b^ |
| 1.91-2.40 | 1.64 (0.74-3.65) ^b^ | 0.225 ^b^ |
| 2.41-2.90 | 1.64 (0.76-3.57) ^b^ | 0.210 ^b^ |
| 2.91-3.40 | 1 (reference) |  |
| >3.40 | 2.75 (1.18-6.45) ^b^ | 0.020 ^b^ |
| CRP | 1.02 (1.01-1.03) ^b^ | < 0.001 ^b^ |
| LDH | 1.00 (1.00-1.01) ^b^ | < 0.001 ^b^ |
| NLR | 1.05 (0.96-1.15) ^b^ | 0.313 ^b^ |

Abbreviations: LDH, lactate dehydrogenase; CRP, C-reactive protein; LDL-C, low-density lipoprotein cholesterol; 95% CI, 95% confidence interval.

a: Multivariate analysis, multivariate logistic regression analysis using 0.6 interval.

b: Multivariate analysis, multivariate logistic regression analysis using 0.5 interval.

Supplementary Table 4. The association between lipid-lowering therapy and severe COVID-19

|  | Lowering lipid therapy | |  | |
| --- | --- | --- | --- | --- |
|  | No | Yes | p value | |
| LDL-C(Severe%) |  |  | |  |
| ≤1.60 | 12 (24.5%) (n=49) | 6 (75%) (n=8) | | 0.015 |
| Age (mean (SD)) | 44.92 (19.85) (n=49) | 59.75 (12.41) (n=8) | | 0.046 |
|  |  |  | |  |
|  |  |  | |  |
| LDL-C (Severe%) |  |  | |  |
| 1.61-3.4 | 78 (18.8) (n=414) | 23 (42.6) (n=54) | | <0.001 |
| Age (mean (SD)) | 44.93 (15.47)(n=414) | 57.07 (13.08)(n=414) | | <0.001 |
|  |  |  | |  |
|  |  |  | |  |
| LDL-C (Severe%) |  |  | |  |
| >3.40 | 19 ( 30.6) (n=62) | 3 ( 21.4) (n=14) | | 0.718 |
| Age (mean (SD)) | 44.97 (14.20) (n=62) | 53.86 (15.94) (n=14) | | 0.042 |

Supplementary Figure 1


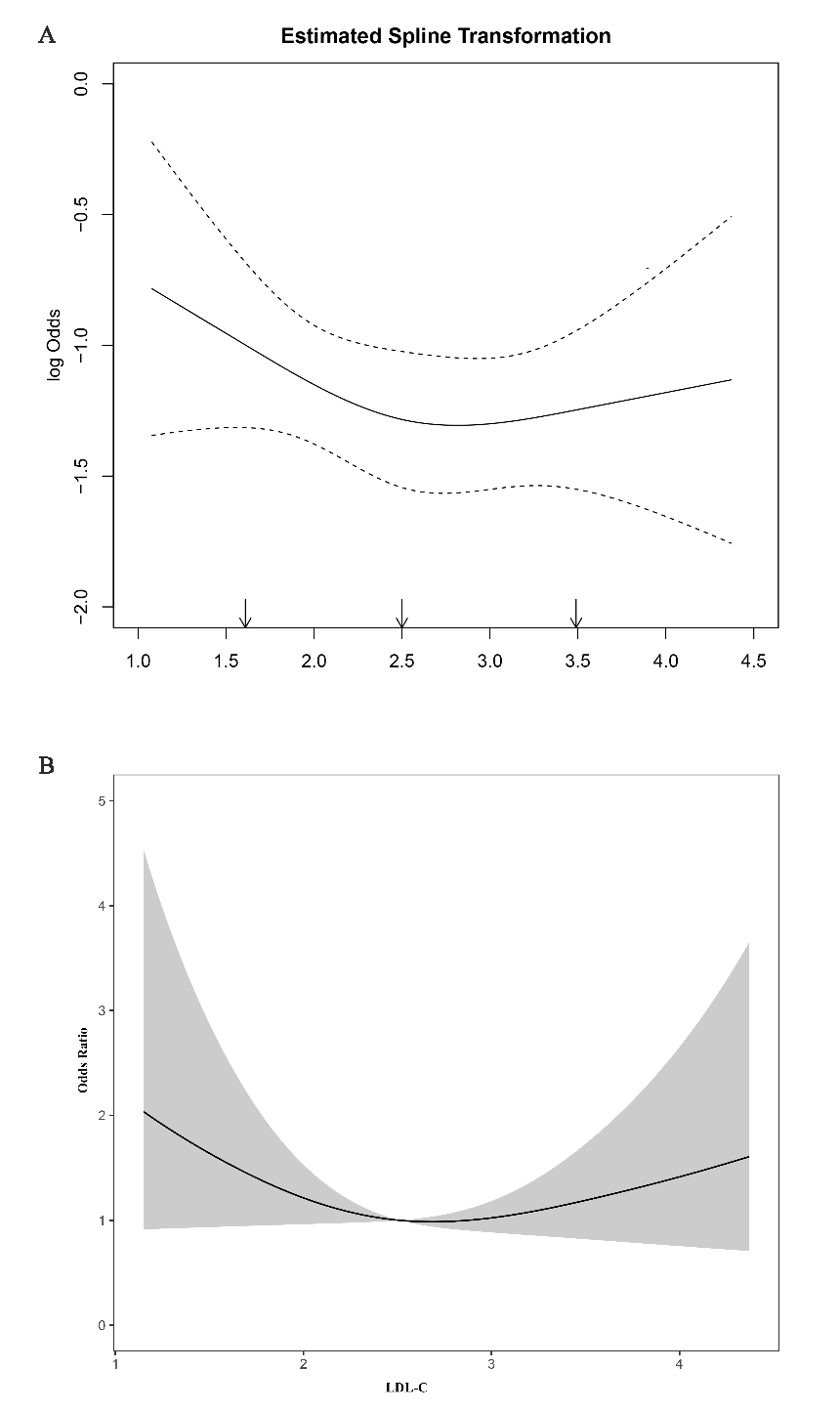


(A) Analyses were conducted using restricted cubic splines, with odds ratios and 95% confidence intervals from univariate logistic regression analysis for LDL-C. (B) Analyses were conducted using restricted cubic splines, with odds ratios and 95% confidence intervals from multivariate logistic regression analysis after adjusted for age, gender, CRP, LDH and NLR.

Supplementary Figure 2


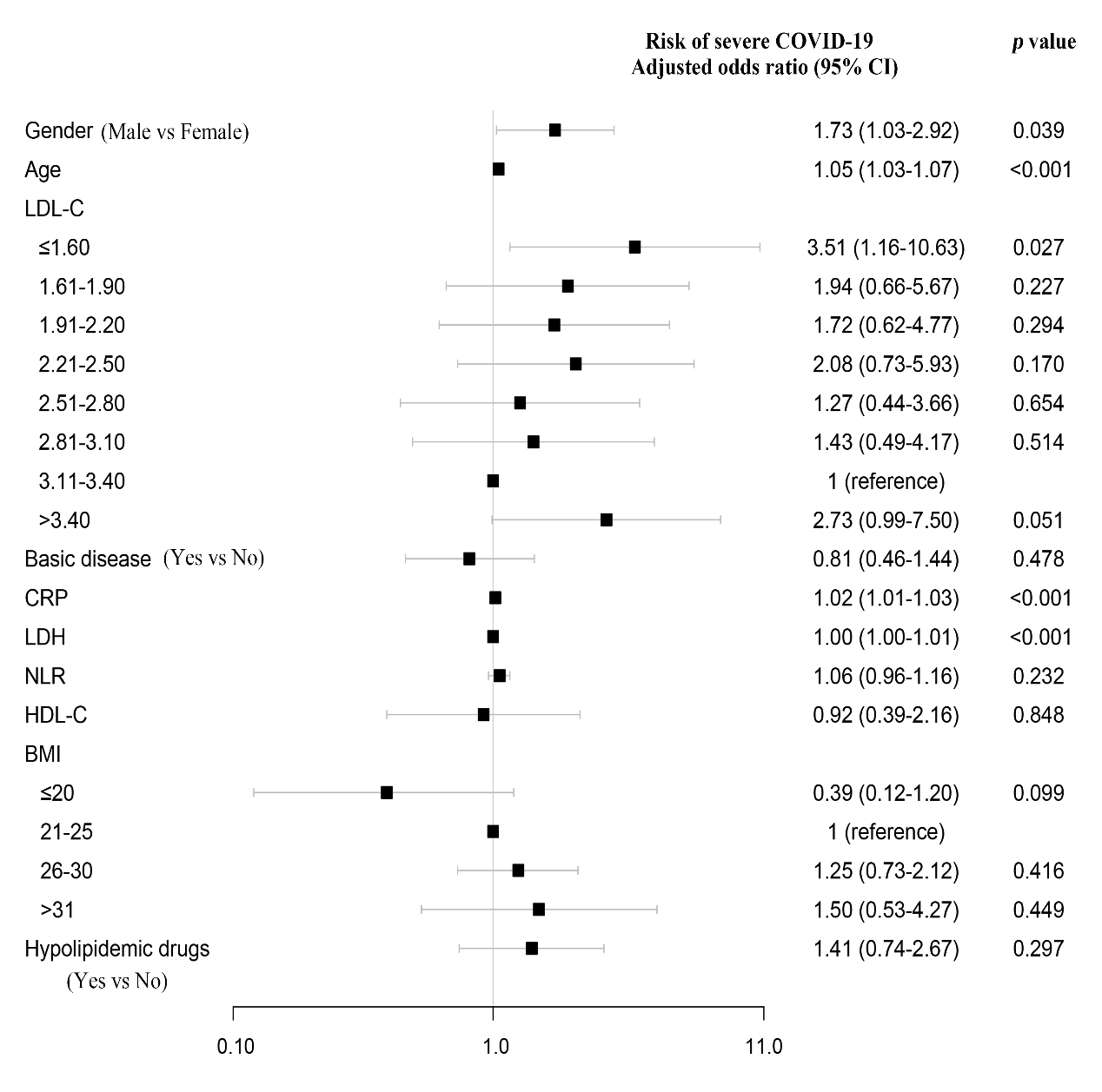


LDL-C and risk of severe COVID-19 using multivariate logistic regression analysis using 0.3 mmol/L categories of LDL-C.

Supplementary Figure 3


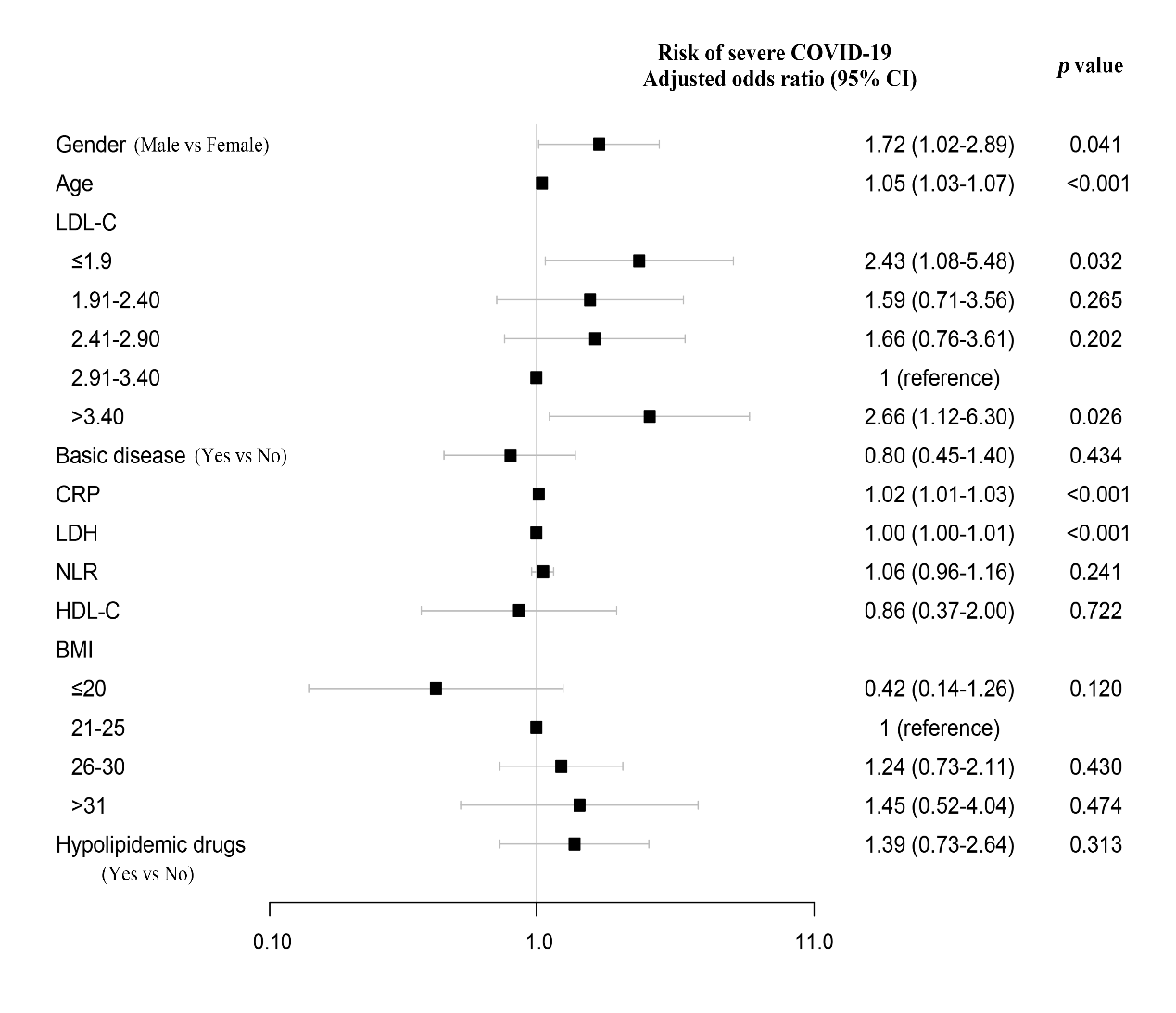


LDL-C and risk of severe COVID-19 using multivariate logistic regression analysis using 0.5 mmol/L categories of LDL-C.

Supplementary Figure 4


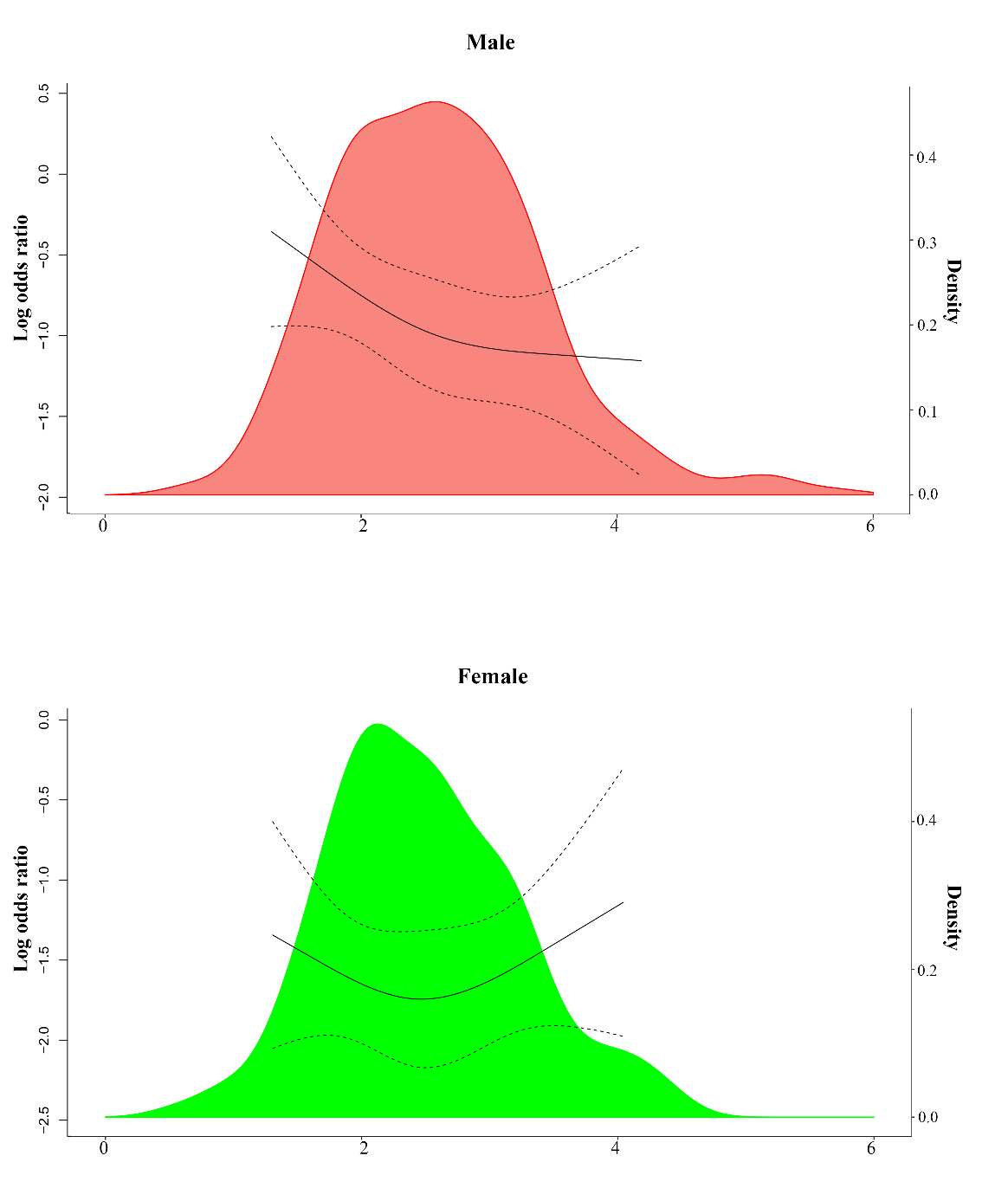


Analyses were conducted using restricted cubic splines, with odds ratios and 95% confidence intervals from univariate logistic regression analysis.
